# Bleomycin electrosclerotherapy (BEST) for the treatment of vascular malformations. An International Network for Sharing Practices on Electrochemotherapy (InspECT) study group report

**DOI:** 10.2478/raon-2023-0029

**Published:** 2023-06-21

**Authors:** Tobian Muir, Giulia Bertino, Ales Groselj, Lakshmi Ratnam, Erika Kis, Joy Odili, Ian McCafferty, Walter A Wohlgemuth, Maja Cemazar, Aljosa Krt, Masa Bosnjak, Alessandro Zanasi, Michela Battista, Francesca de Terlizzi, Luca G Campana, Gregor Sersa

**Affiliations:** Department of Reconstructive Plastic Surgery, James Cook University Hospital, Middlesbrough, United Kingdom; Department of Otolaryngology Head Neck Surgery, University of Pavia, Istituto di Ricovero e Cura a Carattere Scientifico (IRCCS) Policlinico San Matteo Foundation, Pavia, Italy,; Department of Otorhinolaryngology and Cervicofacial Surgery, University Medical Centre Ljubljana, Ljubljana, Slovenia; Faculty of Medicine, University of Ljubljana, Ljubljana, Slovenia; Department of Interventional Radiology, St George's University Hospitals NHS Foundation Trust, London, United Kingdom; Department of Dermatology and Allergology, University of Szeged, Szeged, Hungary; Department of Plastic Surgery, St. Georges University Hospitals NHS Trust, London, United Kingdom; Birmingham Women's and Children's Hospital NHS Foundation Trust, Birmingham, United Kingdom; Universitätsklinik und Poliklinik für Radiologie, Universitätsmedizin Halle, Halle, Germany; Department of Experimental Oncology, Institute of Oncology Ljubljana, Ljubljana, Slovenia; Faculty of Health Sciences, University of Primorska, Slovenia; Department of Otorhinolaryngology, Izola General Hospital, Izola, Slovenia; IGEA S.p.A., Clinical Biophysics Lab. Carpi, Modena, Italy; Department of Surgery, Manchester University NHS Foundation Trust, Manchester, United Kingdom; Faculty of Health Sciences, University of Ljubljana, Ljubljana, Slovenia

**Keywords:** vascular malformations, bleomycin electrosclerotherapy, bleomycin, electrochemotherapy

## Abstract

**Background:**

Biomedical applications of electroporation are expanding out of the field of oncology into vaccination, treatment of arrhythmias and now in the treatment of vascular malformations. Bleomycin is a widely used sclerosing agent in the treatment of various vascular malformations. The application of electric pulses in addition to bleomycin enhances the effectiveness of the drug, as demonstrated by electrochemotherapy, which utilizes bleomycin in the treatment of tumors. The same principle is used in bleomycin electrosclerotherapy (BEST). The approach seems to be effective in the treatment of low-flow (venous and lymphatic) and, potentially, even high-flow (arteriovenous) malformations. Although there are only a few published reports to date, the surgical community is interested, and an increasing number of centers are applying BEST in the treatment of vascular malformations. Within the International Network for Sharing Practices on Electrochemotherapy (InspECT) consortium, a dedicated working group has been constituted to develop standard operating procedures for BEST and foster clinical trials.

**Conclusions:**

By treatment standardization and successful completion of clinical trials demonstrating the effectiveness and safety of the approach, higher quality data and better clinical outcomes may be achieved.

## Classification of vascular malformations

Vascular malformations are a rare condition caused by abnormally developed blood vessels. They can occur anywhere in the body and range from simple and benign to complex conditions, with an incidence of around 1.5% in the general population.

The latest and most used categorization is the International Society for the Study of Vascular Anomalies (ISSVA) classification (Table 1).^1^ This classification divides vascular anomalies into two main categories: tumors (true proliferative neoplasms) and malformations (morphogenetic defects). These two categories are further subcategorized: tumors are divided into benign, locally aggressive/borderline and malignant, whereas malformations are subdivided into simple, combined or associated with other anomalies. Clinically, vascular anomalies can also be divided into low-flow and high-flow malformations.^2^

**TABLE 1.** International Society for the Study of Vascular Anomalies (ISSVA) classification for vascular anomalies 2018

	**VASCULAR TUMORS**		**VASCULAR MALFORMATIONS**	

**Benign**	**Locally aggressive**	**Malignant**	**Simple**	**Combined**
Infantile hemangioma	Kaposiform hemangioendothelioma		Capillary malformation (CM)	CVM, CLM
Congenital hemangioma	Retiform hemangioendothelioma		Lymphatic malformation (LM)	LVM. CLVM
Tufted hemangioma	PILA. Dabska tumor	Epithelioid hemangioendothelioma	Venous malformation (VM)	CAVM
Spindle-cell hemangioma	Composite hemangioendothelioma	Angiosarcoma	Arteriovenous malformation (AVM)	
Epithelioid hemangioma	Kaposi sarcoma		Arteriovenous fistula	CLAVM
Pyogenic granuloma				

AVM = arteriovenous malformation; CAVM = capillary arteriovenous malformation; CLAVM = capillary lymphatic arteriovenous malformation; CLM = capillary lymphatic malformations; CLVM = capillary lymphatic venous malformation; CM = capillary malformation; CVM = capillary venous malformations; LM = lymphatic malformation; LVM = lymphatic venous malformation; PILA – papillary intralymphatic angioendothelioma; VM - venous malformation

## Treatment of vascular malformations

Current approaches vary depending on the type and anatomical location of the vascular malformation. Treatment options include observation, sclerotherapy, laser therapy, embolization, and surgery.^3,4^ Observation is recommended for asymptomatic superficial or low-flow malformations that pose no immediate risk to the patient and are stable in size. Sclerotherapy involves the injection of sclerosing agents, such as bleomycin, or other agents (pingyangmycin, absolute ethanol, ethanolamine oleate, polidocanol, doxycycline, cyanoacrylate, sodium morrhuate and sodium tetradecyl sulfate (STS)).^5,6^ Laser therapy is used to treat superficial vascular malformations and involves the use of a laser to heat the affected area and reduce vessel size. Embolization is a minimally invasive procedure in which small particles, metal coils, or solidifying liquid agents are injected into the malformation to block the flow of blood and reduce its size. This treatment is typically used for high-flow malformations. Surgery may be necessary for high-flow malformations that are difficult to treat with the other methods mentioned. Depending on the size and anatomical location, the surgeon may only remove part of the lesion.^7^

## The use of bleomycin combined with electroporation (electrochemotherapy) in oncology

There are several **biomedical applications of electroporation**. Reversible and irreversible electroporation are distinguished by the amplitude, timing and number of electric pulse applications. Irreversible electroporation is based on irreversible disruption of the cell membrane causing destabilization of cell physiology to the extent that cells die either by apoptosis, necrosis or even immunogenic cell death.^8^ In contrast, reversible electroporation does not cause cell death but temporarily disrupts the cell membrane in such a way that it becomes permeable for molecules that have hampered transport through the membrane.^9,10,11,12^ This phenomenon can be exploited for enhanced drug, DNA or RNA delivery into cells. If we deliver nucleic acids, it is called gene electrotransfer, which can be used for cancer immune-gene therapy; for example, if introduced, DNA or mRNA encodes immunomodulatory molecules. It can also be used for vaccination purposes.^13,14^ The biomedical applications of gene electrotransfer can be used in the treatment of cancer, as well as other diseases, including vaccination for infectious diseases such as SARS CoV-2 virus.^15^ In the case of electrochemotherapy, electroporation is used to enhance the delivery of cytotoxic molecules for cancer treatment. The principle is to inject cytotoxic drugs such as bleomycin or cisplatin into the cancer patient and apply electric pulses at the site of the tumor, where enhanced drug uptake is desired.^16^ Therefore, electrochemotherapy is a local treatment since drug cytotoxicity is enhanced only at the site of electric pulse applications. This approach is being used widely in the treatment of either cutaneous tumors or deep-seated tumors in internal organs, such as liver and pancreas.^16^ Several electrodes were designed that are best suited for the delivery of electric pulses to specific anatomical locations. Due to its simple principle, i.e., the use of highly cytotoxic drugs and the application of electric pulses for its enhanced cytotoxicity, electrochemotherapy is effective on tumors of different histological origins. Its objective response rate ranges between 70–80%.^17^ Electrochemotherapy is listed in many national and international guidelines as a local ablative therapy and is applied in more than 200 centers across Europe.^16^

## Electrochemotherapy mechanisms of action

There are three underlying **mechanisms of electrochemotherapy**. The first is enhanced drug delivery to tumor cells, which die due to the cytotoxicity of the drugs, either by apoptosis or necrosis. This is predominantly related to the drug used and its mode of action. Bleomycin, for example, induces mitotic cell death, which induces slow resolution of the tumor mass. Experimental data on tumors in mice demonstrate that the drug concentration in tumor cells after electroporation is increased 10 times or more, depending on the tumor type, in the case of bleomycin electrochemotherapy.^18^

The second mechanism is the induction of the immune response due to immunogenic tumor cell death induced by the drug. It is well known that certain ablative therapies induce immunogenic cell death that can attract and boost the immune response of the organism. This mechanism has been described in radiation therapy, thermal ablative techniques, and electrochemotherapy.^10,12,19^ Several groups have investigated the role of immunogenic cell death in the effectiveness of electrochemotherapy. Now, the experimental data indicate that the response of the tumors varies depending on the immunogenicity of the tumors; more immunogenic tumors respond better to electrochemotherapy than less immunogenic tumors, which was linked to more pronounced immunogenic cell death after electrochemotherapy.^10,12,20^ Additionally, clinical data on the treatment of melanoma demonstrate that local treatment of cutaneous metastases can boost or interact with treatment using immune checkpoint inhibitors, such as pembrolizumab.^21^ Patients treated with both electrochemotherapy and immune checkpoint inhibitors had lower disease progression rates and longer survival than those who received pembrolizumab only.

The third mechanism is the vascular disrupting effect of electrochemotherapy. In early preclinical research, it was established that the application of electric pulses only temporarily abrogates blood flow within tumors. This phenomenon was termed vascular lock and lasts less than an hour.^22,23^ Furthermore, the effect is enhanced when the drug is present during application of the electric pulses. Investigations have shown that it results in vascular disruption that occurs within hours in tumors. Endothelial cells start to die, blood flow is obstructed, and secondary tumor cell death is induced within days due to induced tumor hypoxia.^24,25,26,27^ The phenomenon is predominantly confined to the tumor vasculature, sparing the normal vasculature around the tumors. The reason for this is because of the high proliferation rate of endothelial cells in tumors compared to the vasculature in normal tissues, where the endothelial proliferation rate is very slow. The vascular disrupting effect of electrochemotherapy is not fully understood. To date, we do not know in what proportion this vascular disrupting effect contributes to the overall effectiveness of electrochemotherapy in specific tumor types. We know that it is dependent on the distribution and extent of tumor vascularization, and better vascularized tumors respond better to electrochemotherapy.^28,29^ Preclinical data also indicate that tumor perfusion influences the effectiveness of the treatment, with well perfused tumors showing an improved response.^30^

## Combination of bleomycin with reversible electroporation for treatment of vascular malformations

**FIGURE 1.**
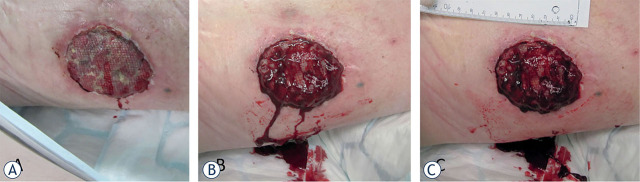
Treatment of vascularized melanoma metastasis by electrochemotherapy. **(A)** Highly vascularized tumor before treatment. **(B)** Bleeding due to electrode insertion after application of electric pulses to the tumor. **(C)** Bleeding stopped immediately after electric pulse application.

Electrochemotherapy is safely applied to palliate bleeding cutaneous metastases and treat vascular tumors e.g., Kaposi sarcoma, superficial angiosarcoma^31,32,33,34^ and highly vascularized liver metastases.^35,36^ Bleeding after the insertion of needle electrodes quickly stops due to vascular lock.^37^ Additionally, both superficial and liver tumors themselves also do not bleed after electrochemotherapy due to the vascular disrupting effect. These observations indicate that electrochemotherapy indeed exerts vascular effects, the abovementioned vascular lock and the vascular disrupting effect. Furthermore, several reports indicate that electrochemotherapy can be safely applied to control or treat bleeding tumors.^37^ Of note, bleeding stops almost immediately after the application of electric pulses; therefore, treatment of bleeding tumors is also one of the indications for electrochemotherapy (Figure 1).

These data support the potential advantage of bleomycin electrosclerotherapy in the treatment of vascular malformations. Bleomycin is already one of the most frequently used sclerotherapy agents in the treatment of these lesions.^5,6^ Therefore, the combination with electric pulses may only add to the effectiveness of bleomycin since it would increase the uptake of the drug into the endothelial lining of the affected blood vessels. In many cases, blood vessels are abnormal and endothelial cell proliferation is higher than that in normal blood vessels.^38^ Therefore, the vasculature in vascular malformations is impaired as it is in tumors, and electrochemotherapy is supposed to be effective in both.

Histological evidence from liver biopsies indicates that venules are more sensitive to electrochemotherapy than arterioles in normal liver parenchyma.^39^ This would indicate that venous malformations would have been more susceptible to BEST.

We recently performed a study on pigs investigating vascular changes in large blood vessels such as the portal vein, inferior vena cava, and lineal vein. In this study, the vessels were directly exposed to electroporation and electrochemotherapy by the application of electric pulses using plate electrodes that embraced the vessels. Electrochemotherapy may temporarily disrupt the endothelial lining and disrupt the vasa vasorum of the vessels (unpublished data). This effect may, however, be a desired one in the treatment of all vascular malformations, specifically because we have not observed thrombi formation in the treated vessels in the pig model.

All this evidence supports the use of bleomycin electroporation in the treatment of vascular malformations, also called bleomycin electrosclerotherapy (BEST). In principle, BEST could be a safe and effective approach for the treatment of vascular malformations; however, more clinical data are needed to confirm this approach. To date, there are only a handful of clinical reports on the treatment of vascular malformations with BEST. Finally, and importantly, the technique needs to be standardized through the development of dedicated standard operating procedures.

## Overview of current clinical BEST reports

Publications on BEST are still limited in number. Table 2 summarizes the clinical studies and case reports published thus far.^40,41,42,43,44,45^ In summary, different types of vascular malformations have been treated, with favorable clinical outcomes. Most of the studies used intralesional bleomycin, either diluted or mixed with lidocaine. Bleomycin dosage varied between studies, but in most reports, it was lower than in traditional sclerotherapy. In addition, the number of treatments required was much lower when BEST was used than when bleomycin was used alone. Drug dosage, the number of treatments needed, and route of drug administration are all aspects that need to be explored to develop recommendations for the future use of BEST.

**TABLE 2.** Clinical studies and case reports using bleomycin electrosclerotherapy^40,41,42,43,44,45^

**Reference**	**Type of malformation**	**N of pts**	**Bleomycin dose and concentration**	**Electrodes used**	**N of pulse applications**	**Response**		**Comment**
McMorrow *et al.*, Br J Oral and Maxillofacial Surg 2017^44^	Venous malformation	1	Reduced dose: 1/3 of the standard dose	Not reported	Not reported	Considerable improvement after 6 unsuccessful sessions with bleomycin		Case report with poor respiratory function
Horbach *et al.*, Dermatologic Surgery 2020^45^	Hypertrophic capillary malformations	5 pts. (out of 20 planned)	0.25 mg or units/cm^3^	Plate & needle	Not reported	7–8 weeks DEROI (changes in colorimetry) Flux in ROI (in Perfusion Units)		Randomized controlled pilot trial
Dalmady *et al.,* Pediatrics 2020^43^	Lymphatic malformation	1	0.5 mg/kg (5.4 mg)	Needle	1^st^ session: 68 applications 2^nd^ session: 74 applications	63% growth-corrected volume decrease. No recurrence at 18 months Follow up		Case report
Wohlgemuth *et al.*, Journal of Vascular Surgery 2021^40^	Venous malformations	17 pts. (20 lesions)	Calculated based on the size of the lesion. C = 0.25 mg/mL Intralesional injection (25% concentration of standard bleomycin sclerotherapy)	Needle & finger	Not reported	3-month post-therapy Changes in volume MRI: Volume reduction,%:		
						> 90%	9 lesions	Retrospective observational case study
						> 70% in < 90%	6 lesions	
						> 50% in < 70%	2 lesions	
						< 35%	2 lesions	
						No response	1 lesion	
Kostusiak *et al*., Dermatologic Surgery 2022^42^	Various vascular malformations	30 pts. VM: 17 AVM: 3 CVM LM: 2 Mixed: 2	Calculated based on the size of the lesion. Bleomycin mixed with 1 mL plain 1% lidocaine Dose not reported	Needle & finger	Not reported	17 Complete Response 7: significant improvement 1: moderate improvement 1: minor response 1: no response 3: active follow up		Prospective observational case study Electrosclerotherapy offered to non-responding patients to standard bleomycin
Krt *et al*., Front Oncol 2022^41^	Arteriovenous malformation	1	750 IU BLM intralesional	Plate	15	CR 18 months after BEST		Case report

AVM = arteriovenous malformation; BLM = bleomycin; CVM = capillary venous malformations; VM - venous malformation; LM = lymphatic malformation;

Another relevant aspect of BEST is the application of electric pulses. Due to the blood accumulated in the malformation, the electrical conductivity of the treated tissue is high. Therefore, it is assumed that the coverage of the target lesion with electric pulses does not need to be so strict as in electrochemotherapy. In this regard, some clinicians who perform BEST on patients report that fewer applications of electric pulses to the lesions are needed. This aspect that requires clarification in future studies.

Studies on BEST should also report the number and amplitudes of pulse applications and the electrical parameters (current). The predominantly used generator is from one producer, and varying electrodes specific to this generator are used. Usually, in each electric pulse application 8 pulses of 1000–1300 V/cm in frequency 5 kHz are applied. Since different electrodes are used for different clinical situations, the reports should describe which electrodes were used. Furthermore, when standard operating procedures for BEST will be prepared, recommendations for the use of specific types of electrodes should also be prepared.

## BEST standardization

To date, there are no standardized guidelines for BEST. As a result, each center applies the treatment according to local protocols and clinical experience. Therefore, standardization of some procedural aspects is needed.

**FIGURE 2.**
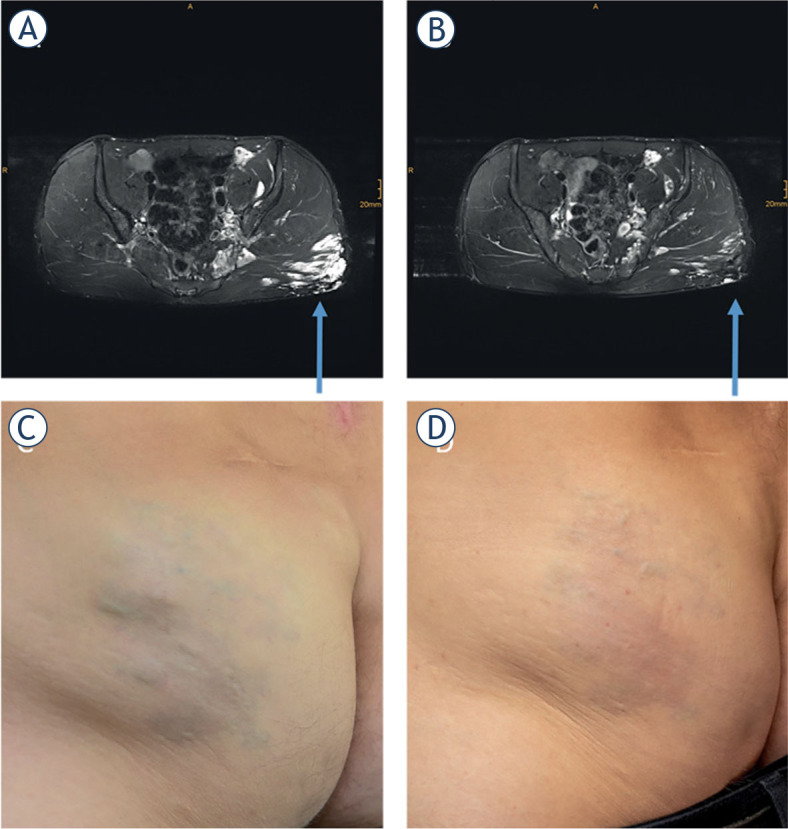
Patient treated by BEST. Axial T2-weighted, fat-saturated MRI with hyperintense (arrow) gluteal venous malformation before treatment **(A)**. Axial T2-weighted, fat-saturated MRI of the same region 4 months after treatment. The main part of the venous malformation is occluded **(B)**. Photography before **(C)** and after the treatment **(D)**.

The spread of a new technology depends on its **safety**. Safety aspects have already been corroborated, since there are already some reports of significant morbidity with bleomycin only, but not with BEST treatment.^46^ We must be aware that cytotoxic drugs are used for the treatment of benign disease, that can be spilled and are sometimes also used in very young patients. This safety aspect is also related to the experience in image-guided bleomycin and electric pulse application (Figure 2).

Currently, BEST must be practiced in the framework of clinical studies, where **patient referral and suitability for BEST treatment need to be defined**. The patients need to be informed about other treatment options and their suitability for the treatment evaluated by a multidisciplinary team (MDT).

It is expected that in the early stage of development, BEST will be considered in recurrences after previous treatments, whereas subsequently more precise indications for specific types of vascular malformations will be individuated.

Other relevant procedural aspects that need to be clarified include drug injection, questions of dosage, the need for general anesthesia, electrode placement and delivery of electric pulses.

Exclusion criteria need to be defined as well, such as pregnancy, lactation and allergy or hypersensitivity to bleomycin, or abnormal respiratory parameters.

The application of electric pulses, especially with needle electrodes, is painful. Therefore, **local or general anesthesia is needed.** In this regard, the experience accumulated with electrochemotherapy can be informative. Generally, the choice of the type of anesthesia is at the discretion of each center. However, there is a recent report that continuous intravenous sedation is also an option that requires less anesthetic and is much shorter in time.^47^

Another important issue is the **route of bleomycin injection**. In electrochemotherapy, the preferred route is intravenous injection; however, in BEST treatment, this is a limited option since intravenous sclerotherapy with bleomycin is not the standard of care. Instead, direct injection is preferred, possibly under image guidance and potentially with a lower dose to avoid systemic toxicity.

In electrochemotherapy, the **bleomycin dose** in intravenous or intratumoral administration is defined.^48^ In elderly patients older than 65 years or with renal impairment, the intravenously administered dose can be decreased by 1/3.^48^ In BEST treatment, the dose can be substantially decreased. The lowest effective dose of bleomycin for BEST treatment still needs to be established. The concentration of bleomycin in the solution needs to be standardized.

Another peculiar aspect is that the **volume of the drug solution** is dependent on the type and volume of the malformation. Furthermore, it depends on whether bleomycin is diluted either with foam, lidocaine or contrast agent and whether there is drainage from the malformation that needs to be stopped, either by compression or intravascular techniques. By all means, the concentration and volume of the drug injected needs to be recorded and reported. The dose and route of administration may also differ in the case of fast or slow flow malformations. The treatment can be repeated up to a cumulative dose for bleomycin of 400 000 IU.^48^

The **interval between the intralesional bleomycin injection and the application of electric pulses** needs to be short, while the drug is present in the treated tissue. The one-minute interval would be enough. A similar situation is also observed in electrochemotherapy, where the time interval is different when bleomycin is injected either intratumorally or intravenously. In the case of intravenous injection, the time interval is 8 minutes, and after intratumoral injection, the time interval is just 1–3 minutes.^48^ The **choice of the electrodes** used is dependent on many factors. The type, location and size of the malformation are the most important factors. Needle electrodes with shorter or longer needles in fixed geometry are available. Some centers have concerns about so-called hexagonal electrodes since they deliver many electric pulses between the needles with a high risk of skin hyperpigmentation at the puncture sites. Some malformations require so-called variable geometry electrodes. These are single long needle electrodes that can be placed separately and can cover deeper mostly subfascial malformations.

Vascular malformations **do not need to be covered entirely with electric pulses,** in contrast to electrochemotherapy. In the case of BEST, damage to the endothelium and vessels needs to be done throughout the malformation but not necessarily with dense and complete coverage of the whole lesion, since the goal is to improve the symptoms more than eradicating the lesion. In this way, the risk of tissue swelling and mucosal ulceration could be reduced.

BEST treatment can be performed as a day case procedure unless pain, bleeding or swelling is anticipated. Generally, follow-up is recommended at approximately three-month intervals.

These are recommendations and considerations for BEST treatment according to the experiences gained by the authors of this manuscript. The members of this working group will continue to share experiences and discuss results to identify the procedural aspects associated with the best results. When a more formal consensus is reached, we will propose our best practice in the form of standard operating procedures (SOPs).

## Role of InspECT in BEST applications

InspECT is an international network of 42 clinical centers using electrochemotherapy for the treatment of cancer. This is the largest group of experts on electroporation-based treatments. Some of them are acquainted with BEST and report a positive experience as other external centers. Together, these centers form a network that can promote BEST worldwide. A dedicated working group for vascular malformations has been formed within InspECT. This group will promote clinical studies with BEST and seek collaboration with other centers. This paper aims to raise interest and awareness in the treatment of vascular malformations with BEST and provide an overview of the current status of development of this approach.

## Future directions and conclusions

The number of clinicians using BEST to treat vascular malformations is growing. Due to the first and positive experiences in various vascular malformations, BEST application is being practiced in an increasing number of centers throughout Europe and the UK. This article summarizes the rationale and underlying mechanisms of BEST, along with the initial clinical experiences. Additionally, it highlights the main controversial procedural aspects and the need for dedicated SOPs.
